# Earlier onset and slower heartwood investment in faster-growing trees of African tropical species

**DOI:** 10.1093/aob/mcad079

**Published:** 2023-07-06

**Authors:** Chadrack Kafuti, Romain Lehnebach, Nils Bourland, Hans Beeckman, Joris Van Acker, Nestor K Luambua, Jan Van den Bulcke

**Affiliations:** UGent-Woodlab, Laboratory of Wood Technology, Department of Environment, Ghent University, Coupure Links 653, 9000 Gent, Belgium; Service of Wood Biology, Royal Museum for Central Africa, Leuvensesteenweg 13, 3080 Tervuren, Belgium; Faculty of Agricultural Sciences, Department of Natural Resources Management, University of Kinshasa, 117 Kinshasa XI, Democratic Republic of the Congo; UGent-Woodlab, Laboratory of Wood Technology, Department of Environment, Ghent University, Coupure Links 653, 9000 Gent, Belgium; CIRAD, UMR Ecologie des Forêts de Guyane (EcoFoG), AgroParisTech, CNRS, INRA, Université Des Antilles, Université de Guyane, 97310 Kourou, France; Service of Wood Biology, Royal Museum for Central Africa, Leuvensesteenweg 13, 3080 Tervuren, Belgium; Center for International Forestry Research, Situ Gede, Sindang Barang, Bogor (Barat) 16115, Indonesia; Service of Wood Biology, Royal Museum for Central Africa, Leuvensesteenweg 13, 3080 Tervuren, Belgium; UGent-Woodlab, Laboratory of Wood Technology, Department of Environment, Ghent University, Coupure Links 653, 9000 Gent, Belgium; Service of Wood Biology, Royal Museum for Central Africa, Leuvensesteenweg 13, 3080 Tervuren, Belgium; Faculté des sciences Agronomiques, Université Officielle de Mbujimayi, Mbujimayi, Democratic Republic of Congo; UGent-Woodlab, Laboratory of Wood Technology, Department of Environment, Ghent University, Coupure Links 653, 9000 Gent, Belgium

**Keywords:** Afrormosia, Congo Basin, heartwood formation, hydraulic functioning, *Pericopsis elata*, sapwood, tree growth, tropical forests

## Abstract

**Background and Aims:**

Heartwood plays an important role in maintaining the structural integrity of trees. Although its formation has long been thought to be driven solely by internal ageing processes, more recent hypotheses suggest that heartwood formation acts as a regulator of the tree water balance by modulating the quantity of sapwood. Testing both hypotheses would shed light on the potential ecophysiological nature of heartwood formation, a very common process in trees.

**Methods:**

We measured quantities of heartwood and sapwood, xylem conduits and the width and number of growth rings on 406 stems of *Pericopsis elata* with ages ranging from 2 to 237 years. A subset of 17 trees with similar ages but varying growth rate were sampled in a shaded (slower-growth) site and a sun-exposed (faster-growth) site. We used regression analysis and structural equation modelling to investigate the dynamics and drivers of heartwood formation.

**Key Results:**

We found a positive effect of growth rate on the probability of heartwood occurrence, suggesting an earlier heartwood onset in faster-growing stems. After this onset age, heartwood area increased with stem diameter and age. Despite the similar heartwood production per unit stem diameter increment, shaded trees produced heartwood faster than sun-exposed trees. Tree age and hydraulics showed similar direct effects on heartwood and sapwood area of sun-exposed trees, suggesting their mutual role in driving the heartwood dynamics of sun-exposed trees. However, for shaded trees, only tree hydraulics showed a direct effect, suggesting its prominent role over age in driving the heartwood dynamics in limited growing conditions. The positive relationship between growth rate and maximum stomatal conductance supported this conclusion.

**Conclusions:**

Heartwood area increases as the tree ages, but at a slower rate in trees where water demand is balanced by a sufficient water supply. Our findings suggest that heartwood formation is not only a structural process but also functional.

## INTRODUCTION

Sapwood has a key ecophysiological function. As the xylem part containing active living cells, sapwood is involved in vital functions, such as sap transport to the leaves, storage of water and carbohydrates and mechanical support. Through these functions and the associated maintenance cost induced by respiration, sapwood dynamics drive numerous structural adjustments in the trees. Sapwood is an important determinant of primary (i.e. height growth rate) and secondary (i.e. diameter or basal area growth rate) tree growth (Moreno Chan *et al.*, 2013; Olson *et al.*, 2014; van der Sande *et al.*, 2015). Primary growth induces an increase of the xylem pathway and resistance to sap transport, causing constraints on the plant water balance, increasing the risk of catastrophic xylem cavitation (Fulton *et al.*, 2014). To maintain a balance, several adjustments occur in the tree, such as maintaining a higher sapwood area (Delzon *et al.*, 2004). However, a similar sapwood area can be associated with different abilities to supply water, owing to differences in the xylem structure of the sapwood. The theoretical model of West, Brown and Enquist (WBE model; West *et al.*, 2009) proposes that a proper tapering of xylem conduits can be a sufficient adjustment, leading to a full compensation of the effect of height. If this adjustment is not sufficiently assured, trees can experience hydraulic dysfunction. Moreover, the recovery of embolized vessels, which is crucial for tree survival under hydraulic constraints, is strongly controlled by sapwood non-structural carbohydrates (Yoshimura *et al.*, 2016). However, the quantity of sapwood in trees is not static, but is continually converted into heartwood (i.e. heartwood formation) from a certain size and age onwards and at a certain rate, while incremented through secondary growth. Despite their importance in tree functioning, the timing, dynamics and drivers of heartwood formation are poorly elucidated, especially for tropical tree species.

The timing of heartwood formation is estimated through the age or size of the tree at the onset of heartwood formation (i.e. age of heartwood onset). Previous studies have reported a varying age of heartwood onset between species. The age of heartwood onset was estimated at 6 years for *Pinus banksiana* (Yang and Hazenberg, 1991*a*), 30 years for *P. canariensis* (Climent *et al.*, 2002), 50–67 years for *P. clausa*, *P. pungens*, *P. serotina* and *P. ponderosa* (Koch 1972), and 5 years for *Populus temuloides* (Yang and Hazenberg, 1991*b*). Varying ages of heartwood onset were also reported for the same species, especially in *Pinus pinaster* (Pinto *et al.*, 2004; Esteves *et al.*, 2005; Knapic and Pereira, 2005) and *Pinus sylvestris* (Hägglund, 1951; Björklund, 1999; Mörling and Valinger, 1999). For tropical species, similar studies are very rare. In *Tectona grandis*, Fernández-Sólis *et al.* (2018) reported an onset age of 2 years, whereas Moya *et al.* (2014) reported onset ages ranging from 4 to 6 years, and Hao *et al.* (2022) reported an onset age of 7 years. The reasons of these large variations in the timing of heartwood onset are still unclear, but some studies have suggested the role of environmental conditions, tree stature or forest management practices as potential drivers. In *Pinus sylvestris*, Hägglund (1951) found a similar age of heartwood onset for all trees within a specific geographical location but higher ages in higher-latitude locations and associated this with the effect of the mean maximum temperature. For the same species, some pioneer studies reported that trees with a broad canopy and growing in warmer and drier regions delayed their heartwood onset (Pilz, 1907; Harris, 1954). The most likely factor driving these variations in the age of heartwood onset would be the growth rate. Faster growth is associated with a greater water use. When water use reaches a level that is not consistent with water availability, a decrease in the moisture content of the innermost sapwood occurs, leading to the formation of heartwood (Harris, 1954; Bamber, 1976). We therefore hypothesize an early onset of heartwood in fast-growing stems, which aligns with the assumption of heartwood formation as an adaptation to regulate the stem conductance capacity, hence the water use of the tree (Stokes and Berthier, 2000; Medhurst and Beadle, 2002; Tyree and Zimmermann, 2002; Moreno Chan *et al.*, 2013). As a consequence, tree hydraulic requirements would be as an important driver of heartwood dynamics.

The dynamics of heartwood formation refer to the amount of heartwood produced per year or per unit of size increase. Previous studies have reported significant relationships between heartwood quantities and tree or stem age or size. But the type of the relationship varies between species and as a function of the variable used to express the quantity of heartwood. Using the number of growth rings included in heartwood, studies reported a higher and constant rate of heartwood production after a certain age. In *Pinus banksiana*, heartwood is produced at an average rate of 0.57 rings year^−1^ for ages <70 years and 1 ring year^−1^ above this age (Yang and Hazenberg, 1991*a*). A higher and constant rate of heartwood production after a certain age was also reported for *Pinus pinaster* (Pinto *et al.*, 2004; Knapic and Pereira, 2005), *Pinus sylvestris* (Björklund, 1999; Gjerdrum, 2003) and *Picea mariana* (Hazenberg and Yang, 1991). Although relevant in highlighting differences in the ecophysiological strategy of these temperate species, this large variation in the rate of heartwood production between and within species suggests that heartwood production is driven not only by tree age. Similar studies are very rare for tropical species, although some studies using heartwood radius, heartwood area or the proportion of heartwood area exist for both temperate (Berthier *et al.*, 2001; Wang *et al.*, 2010; Moreno Chan *et al.*, 2013) and tropical species (Morais and Pereira, 2007; Fernández-Sólis *et al.*, 2018). In all these studies, the quantity of heartwood was shown to be significantly related to the tree or stem size, although some studies also revealed a significant relationship with tree or stem age (Moya *et al.*, 2014). However, the effect of size on quantity of heartwood includes not only the hydraulic and metabolic components, attributable to the increasing hydraulic requirements and maintenance cost associated with the size increase, but also the effect of age as a result of the changes in tree size as the tree ages. Yet, the exact roles of tree hydraulics and age still need to be disentangled. Studying the heartwood quantities in trees of the same age but of different sizes could help to disentangle the effects of hydraulics and age on heartwood formation and improve our knowledge of the drivers of this process in tropical timber species. This is particularly relevant, not only at the ecophysiological level but also for management and conservation purposes.

Understanding the dynamics and drivers of heartwood formation of timber species can help to maximize the benefit of individual tree harvesting and avoid cutting trees with too low a proportion of heartwood, given that heartwood is more interesting for the timber market than sapwood. Estimating the amount of wood volume used or needed to produce a given product, especially for endangered timber species is also highly relevant for policy implementation.

As an endangered species and one of the most valuable and highly logged timber species in the poorly studied Congo Basin forests, *Pericopsis elata* (Harms) Meeuwen (Fabaceae) is a relevant candidate species for the study of heartwood formation. Populations of this species are decreasing throughout its natural distribution range because of overexploitation, reduced natural regeneration and, potentially, climate change (Kafuti *et al.*, 2022). The last large populations are found in the Democratic Republic of the Congo, in Cameroon and the Republic of Congo (Dickson *et al.*, 2005). The species is commonly harvested for its heartwood, which is known for its high natural durability, mechanical strength and dimensional stability, combined with its widely appreciated decorative value. Unfortunately, little is known about the dynamics of heartwood formation of this species or for any other timber species of the region. To fill this knowledge gap, we investigated the timing, dynamics and drivers of heartwood formation in *P. elata* and examined the assumptions about ageing and hydraulics of heartwood formation in this flagship timber species of the Congo Basin. We were interested specifically in addressing the following research questions:

1. When does *P. elata* start to form heartwood and what triggers this onset timing?
2. What is the rate of heartwood formation?
3. How do tree age and hydraulic requirements affect the heartwood formation?

We examined the role of different variables associated with the hydraulic requirements of the tree, including the xylem conduit size, the total leaf area (*A*_L_; in metres squared) and the maximum stomatal conductance.

## MATERIALS AND METHODS

### Study sites

This study was conducted in the tropical rainforest of the northern Democratic Republic of the Congo. Trees were selected around Kisangani, capital city of the province of Tshopo. Three sites were selected: Yangambi (100 km north-west of Kisangani; 00°48ʹN, 24°29ʹE), Babusoko (62 km south-east of Kisangani; 00°18ʹN, 25°19ʹE) and Babagulu (59 km east of Kisangani; 00°34ʹN, 24°39ʹE). In Yangambi, trees were selected from an old-growth semi-deciduous forest subjected to silvicultural activities in 1938 and from a 45-year-old plantation where trees were planted in an agroforestry system with cacao. In Babusoko and Babagulu, trees were selected in an old-growth unmanaged forest located in the logging concessions 46/11 and 05/18 respectively granted to the Compagnie Forestière et de Transformation.

The three study sites have equatorial climatic conditions. The average (±s.d.) annual precipitation recorded at the meteorological station of Yangambi (1931–2017) is 1811.7 ± 214.8 mm, with an average temperature of 24.94 ± 0.30 °C (Likoko *et al.*, 2019). No distinct dry season is observed, but the region receives <100 mm of rainfall monthly between December and February. Temperatures are high and constant through the year, with the highest values in March (25.50 ± 0.60 °C) and the lowest in July (24.20 ± 0.40 °C). The region is characterized by nutrient-poor and deeply weathered Ferralsols, formed from fluvio-aeolian sediments composed of quartz sand, kaolinite clay and hydrated iron oxides (Doetterl *et al.*, 2015). As part of the Guineo-Congolean regional centre of endemism, the three study sites are covered by semi-deciduous forests (White, 1986) consisting of mixed old-growth forests, characterized by *Scorodophloeus zenkeri* (Fabaceae), monodominant forests of *Gilbertiodendron dewevrei* (Fabaceae) and *Brachystegia laurentii* (Fabaceae), and by old regrowing forests, characterized by species such as *Khaya anthotheca* (Meliaceae), *Entandrophragma cylindricum* (Meliaceae), *P. elata* (Fabaceae) or *Milicia excelsa* (Moraceae) in the dominant stratum (Kafuti *et al.*, 2020).

### Tree selection, sampling and measurements

Between 1960 and 1970, different agroforestry and silvicultural experiments were undertaken in Yangambi to assess the agroforestry potential and the ability of some timber species to regenerate naturally. The agroforestry experiment consisted of using *P. elata* trees as shading trees for cacao cultures, which require shade for their optimal growth. In this experiment, *P. elata* trees grew with a fully sun-exposed crown throughout their lifespan. The silvicultural experiment consisted of eliminating competitive vegetation around young trees of *P. elata* and 12 other light-demanding timber species to stimulate their growth. In addition, complete removal of vegetation on areas covering ≤2000 m^2^ was applied to stimulate the natural regeneration of these species. After the establishment of trees, no further actions were performed to continue maintaining an open canopy. Consequently, the canopy closed after years, and all the new trees grew as shaded or understorey trees throughout their lifespan, with a very limited exposure to sunlight. In this study, we selected 17 individuals of *P. elata* from the two experiments: seven sun-exposed trees (i.e. fully exposed crown) of the agroforestry experiment (called sun-exposed trees hereafter) and ten shaded trees of the silvicultural experiment (called shaded trees hereafter).

**Fig. 1.**
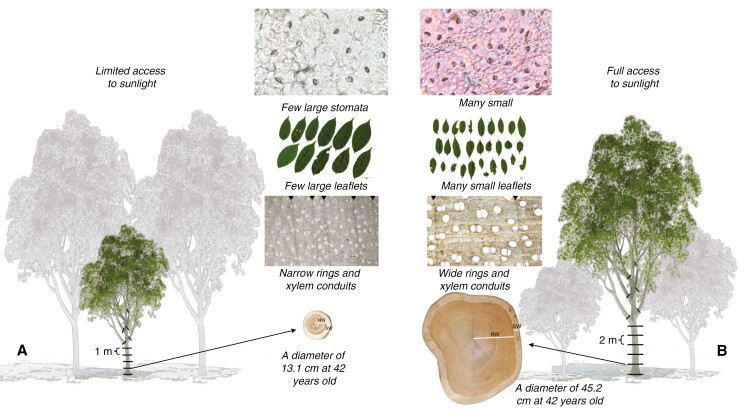
Illustration of the lower growth rate (smaller stem size at similar age), narrower growth rings and xylem conduits, larger leaf size and smaller number of stomata of shaded trees (A) compared with sun-exposed trees (B). Heartwood (HW) and sapwood (SW) are highlighted in the stem discs using white lines, and the boundaries of growth rings are highlighted on the wood microsections using black triangles. Images were made with objective lenses of ×4 for wood microsections and ×40 for stomatal imprints.

On each tree, we measured the diameter at breast height (in centimetres) and the crown radius (in metres) in eight sub-cardinal directions (north, north-west, west, south-west, south, south-east, east and north-east) (Cailliez, 1980). Tree diameter at breast height was measured using a tape at 130 cm height from the ground level or 30 cm above the butt-swelling zone. The crown radius in a specific direction was measured as the distance from the centre of the trunk to the perimeter of the crown using the vertical sighting method (Preuhsler, 1981). Between May and August 2018, the 17 selected trees were felled to quantify their amounts of heartwood and sapwood, their growth rate and their wood and foliar traits. On each felled tree, we measured the total height (*H*; in metres) and the trunk height (*h*; in metres) using a tape. We also measured the stem diameter over bark at constant intervals from the base to the top of the trunk. The selected interval was 1 m for shaded trees and 2 m for sun-exposed trees. At each point where the stem diameter was measured, a wood disc was collected. We also collected all the branches of each tree. On each branch, we measured the base diameter and the total length and collected a stem disc at the base of the branch (Fig. 1). We also collected all the leaves of each branch from each tree. To avoid loss of leaves, branches were harvested from standing trees using the zipline technique performed by professional tree climbers.

To increase the tree size and age coverage in the dataset, additional trees were sampled at Babusoko (11 trees) and Babagulu (125 trees) amongst trees selected for logging by the timber company Compagnie Forestière et de Transformation. These trees were sun exposed. On each of these trees, we measured the diameter at breast height and the trunk height and collected two wood discs at the base and the top of the trunk of all the 11 trees in Babusoko and 37 of the 125 trees in Babagulu. The height from the ground to the location where wood discs were extracted was measured carefully using a tape. Globally, we sampled 153 trees and collected 236 wood discs from the trunk and 166 discs from branches.

### Measurements of foliar and wood traits

Leaves (i.e. leaflets) collected from the 17 trees in the shaded and sun-exposed experiments in Yangambi were first weighted to obtain the total fresh mass of leaves from each branch, then summed to obtain the total green mass of leaves of each tree. On each branch of each tree, we randomly selected leaves corresponding to 10% of the total green mass of leaves of the branch. The selected leaves were counted to obtain the number of leaves, then scanned using an EPSON Expression 11000XL scanner and their surface area determined by image analysis using ImageJ software (Schneider *et al.*, 2012). Finally, the selected leaves were oven dried at 70 °C for 72 h and their dry mass was measured. We calculated the average area (*a*_L_; in centimetres squared), green mass and dry mass of leaves to compute the total number, total area (*A*_L_; in metres squared) and total dry mass of leaves. We measured the stomatal density (*D*_stomata_) and size (*S*_stomata_) and calculated the maximum stomatal conductance to water vapour (*G*_max_; in moles per metre squared per second) on a subset of 15 leaves selected from three branches (a bottom, a middle and a top crown branch). We used the protocol described by Kafuti *et al.* (2020) for the processing of leaf samples and for the measurement and calculation of stomatal variables.

Wood discs were first dried in the open air for 1–2 months, then sanded using grits ranging successively from 40 to 1200. Sanded discs were then scanned at 1200 dpi using an A3 flatbed scanner (Epson Expression 10000 XL; Epson, Nagano, Japan). For each disc, the cross-sectional area of the disc with and without dry bark and the cross-sectional area of heartwood (HWa; in centimetres squared) were determined by image analysis using ImageJ software. Depending on the size of the wood disc, one or several scans were carried out with an overlapping strip of ≥3 cm following Latte *et al.* (2015), then combined to create a mosaic using the plugin ‘MosaicJ’ (Thévenaz and Unser, 2007) in the ImageJ software. The cross-sectional area of sapwood (SWa; in centimetres squared) was calculated as the difference between the cross-sectional area of the disc without bark and the cross-sectional area of heartwood. We then measured the radius of both stem and heartwood in eight equally distributed directions, with a 45° angle from each other. The mean stem radius under bark (*S*_R_; in centimetres) and heartwood radius (HW_R_; in centimetres) were calculated as the quadratic average of the eight radii to account for the commonly observed stem eccentricity of *P. elata*. The mean width of sapwood (SW_R_; in centimetres) was calculated as the difference between the mean radius of the stem and the mean radius of heartwood.

Wood discs were then observed with an iDS UI-3250CP-C-HQ camera (Boston Microscopes, USA) mounted on an Olympus BX51M light microscope (Spach Optics Inc., Rochester, NY, USA). The rings boundaries were marked, and the number of growth rings from pith to bark and the number of rings included in heartwood (HW_rg_) and sapwood (SW_rg_) were counted carefully. *Pericopsis elata* forms annual growth rings, with a thin parenchyma layer produced during the driest months of the year as the ring boundary (de Ridder *et al.*, 2014; Colombaroli *et al.*, 2016). Consequently, we used the number growth rings as an estimate of the cambial age. To account for the top-to-bottom variation in the growth rate of trees, we calculated the growth rate on each stem disc as the ratio of the stem diameter and the cambial age.

From each wood disc from the shaded and sun-exposed trees of Yangambi, we extracted a cubic piece of wood from the outer part of the disc for microsectioning (10–20 µm thickness) using a sliding microtome. The microsections produced were digitized using a Toupview UH-CMOS camera mounted on an Olympus BX51M light microscope, then analysed with ImageJ software to measure the vessel density, vessel area and vessel diameter on ≥30 randomly selected vessels. Because of the elliptical shape of the vessels, two diameters were measured on each vessel, the diameter of the major axis (*a*_*i*_) and the diameter of the minor axis (*b*_*i*_), using the ‘*Ferret diameter*’ option of ImageJ software. Using these two vessel diameters, we calculated the hydraulically weighted vessel diameter (*D*_H_; in micrometres) using the modified formula suggested by Nobel (2009):


$$\begin{array}{l} D_{H}=\sqrt[{4}]{\frac{1}{n}\underset{i=1}{\overset{n}{\sum }}\;\frac{2a_{i}^{3}b_{i}^{3}}{a_{i}^{2}+b_{i}^{2}}} \end{array}$$ (1)

We also calculated the total conductive vessel area (TCVA; in centimetres squared) as the product of the vessel area, vessel density and sapwood area of each stem and calculated the vessel lumen fraction (*F*_V_; as a percentage) and the ratio of TCVA and SWa.

### Statistical analyses

To estimate the difference in the growth rate and hydraulic structure of shaded and sun-exposed trees, we used the Kruskal–Wallis test to compare mean values of hydraulic-related foliar and wood traits measured on shaded and sun-exposed trees. We also used standardized major axis regression (SMA) on ln-transformed variables to compare the slopes of the relationship between stem diameter and age (expressing the dynamics of stem growth) and the relationship between total leaf area and age (expressing the dynamics of water demand).

The diameter and age at heartwood onset were determined as, respectively, the lowest diameter and age of stems exhibiting heartwood, merging all samples from trunks and branches. Threshold values of the diameter and age of heartwood onset were estimated using a modified logistic regression model describing the probability of appearance of heartwood in the stem of *P. elata* as a function of the logarithm of stem diameter (eqn 2) or cambial age (eqn 3):


$$\begin{array}{l} P=\exp\left(a+b\times \log D\right)/\left[1+exp\left(a+b\times \log D\right)\right]\; \end{array}$$ (2)

$$\begin{array}{l} P=\exp\left(a+b\times \log Age\right)/\left[1+exp\left(a+b\times \log Age\right)\right]\; \end{array}$$ (3)

where *P* is the probability of heartwood occurrence, *a* and *b* are the intercept and the slope of the relationship between presence and absence of heartwood, and the logarithm of stem diameter (*D*) or age (*Age*). The inflection point of the fitted response was used as the diameter or age threshold value after which heartwood is present. We tested for the differences between shaded and sun-exposed trees by including site (shaded vs. sun-exposed) in the models (eqn 4). To unravel the source of this between-site difference, we tested for the effect of growth rate (GR), replacing site by growth rate in the logistic models (eqn 5).


$$\begin{array}{l} P=\exp\left(a+b\times \log D+c\times site\right)\;/\left[1+exp\left(a+b\times \log D+c\times site\right)\right]\; \end{array}$$ (4)

$$\begin{array}{l} P=\exp\left(a+b\times \log Age+c\times \log GR\right)\;/\left[1+exp\left(a+b\times \log Age+c\times \log GR\right)\right]\; \end{array}$$ (5)

The dynamics of heartwood formation were evaluated through the relationship between heartwood quantities and stem diameter or stem age. We therefore used regression analysis between heartwood variables (heartwood area, proportion of heartwood area and number of growth rings included in heartwood) and diameter or cambial age. For each relationship, the models with the lowest residual standard error were selected among a series of candidate models. We used the likelihood ratio test to evaluate the significance of the difference in the slope of shaded and sun-exposed trees. Regression analysis was also used to assess the within-tree variation of stem, sapwood and heartwood radius and area and xylem conduit variables (hydraulically weighted vessel diameter and TCVA) within each tree. We therefore evaluated the relationship between these variables and the distance from the tree top. We used SMA on ln-transformed variables. We evaluated the difference between shaded and sun-exposed trees by testing the differences between slopes (called tapering hereafter) of the respective SMAs. We used the same approach to test for the differences in the stem diameter increment (stem diameter vs. cambial age) and annual rate of investment in leaf area (total leaf area as function of cambial age) between sun-exposed and shaded trees. For *D*_H_, we also computed tree-specific tapering values as an indicator of the vascular architecture of the tree (West *et al.*, 1999; Anfodillo *et al.*, 2006).

The drivers of heartwood formation were disentangled through structural equation models describing the direct and indirect effects of age and hydraulic-related variables on the heartwood area at the trunk level or at the sapwood area at the branch level of *P. elata*. We used the TCVA and the total leaf area as the hydraulic-related variables at the trunk and the branch levels, respectively. The TCVA was not calculated on branches because some branches were too small to allow unbiased estimations of the vessel density. The significance of the difference between effects was evaluated using the Wald test (Wald, 1943) from the R package ‘*lavaan*’ (Rosseel, 2012). For all the models, residual analysis was performed to evaluate the validity of the model. All analyses were performed in the software R (R Core Team, 2021).

## RESULTS

### Growth rate and hydraulic structure of shaded and sun-exposed trees

**Fig. 2.**
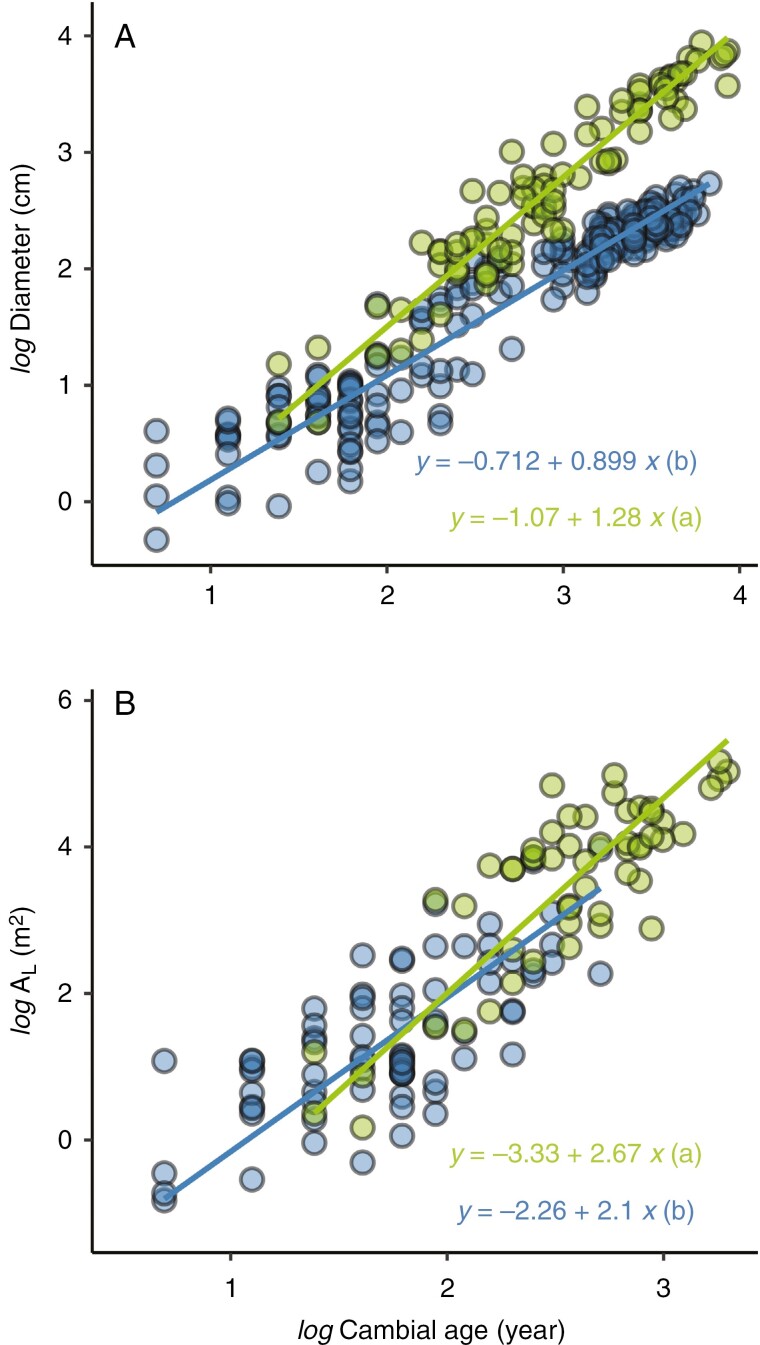
(A) The relationship between stem diameter and cambial age of *Pericopsis elata* reveals a higher growth rate of sun-exposed trees (green) compared with shaded trees (blue). (B) The relationship between the total leaf area (*A*_L_; in metres squared) of branches and cambial age reveals a faster investment in leaf area of sun-exposed trees compared with shaded trees. All 236 discs from the trunk and 166 discs from the branches are included in A and B, respectively.

Shaded and sun-exposed trees differed significantly in their growth rate and hydraulic structure (Fig. 2) at similar ages. For the same age, we found higher stem diameters in sun-exposed trees compared with shaded trees (Fig. 2A). The slopes of the linear relationship between stem diameter and cambial age revealed an average annual stem increment of 0.30 and 1.02 cm year^−1^ for shaded and sun-exposed trees, respectively. This large difference in growth rate resulted in significant differences in tree size and hydraulic-related traits (Table 1). The average diameter at breast height was 11.56 ± 1.04 and 43.03 ± 4.67 cm for shaded and sun-exposed trees, respectively. Sun-exposed trees showed larger vessel sizes and a larger fraction of vessel area and thus larger TCVA than shaded trees. They also showed a higher total leaf area and maximum stomatal conductance than shaded trees, but a lower average leaf area. At the branch level, we found that the total leaf area of a branch increased with the cambial age of the branch. This relationship was significantly different between sun-exposed and shaded trees, with a high annual rate of increase in the total leaf area of sun-exposed trees compared with shaded trees (Fig. 2B).

**Table 1.** Hydraulic-related wood and foliar traits of sun-exposed and shaded trees of *Pericopsis elata*

Trait	Sun-exposed trees (*N* = 10 trees)	Shaded trees (*N* = 7 trees)
*D*_H_ (µm)	123.55 ± 8.81^a^	101.33 ± 9.67^b^
*N*_V_ (mm^−1^)	17.26 ± 2.82^a^	18.06 ± 3.27^a^
*A*_V_ (mm^2^)	0.115 ± 0.017^a^	0.076 ± 0.014^b^
TCVA (cm^2^)	63.35 ± 25.58^a^	5.62 ± 1.90^b^
*F*_V_ (%)	19.88 ± 4.42^a^	13.65 ± 3.41^b^
*T*_conduit_	0.15 ± 0.23^a^	−0.38 ± 0.27^b^
*a*_L_ (cm^2^)	9.73 ± 1.28^b^	33.82 ± 4.98^a^
*A*_L_ of branches (m^2^)	54.83 ± 46.30^a^	6.60 ± 8.63^b^
*A*_L_ of the whole tree (m^2^)	3,070.60 ± 46.30^a^	508.34 ± 8.63^b^
*G*_max_ (mol m^−2^ s^−1^)	1.98 ± 0.32^a^	1.33 ± 0.18^b^

The traits are as follows: hydraulically weighted vessel diameter (*D*_H_), vessel density (*N*_V_), vessel area (*A*_V_), total conductive vessel area (TCVA), vessel lumen fraction (*F*_V_), tapering of xylem conduit along the tree trunk (*T*_conduit_), mean leaf area (*a*_L_), total leaf area (*A*_L_) of individual branches and of the whole tree, and maximum stomatal conductance (*G*_max_). Results of the Kruskal test comparing the mean value of the traits between sun-exposed and shaded trees are also provided through the letters next to the mean ± s.d. values, with different letters indicating significant differences.

### *Size and age of heartwood onset in* P. elata

**Fig. 3.**
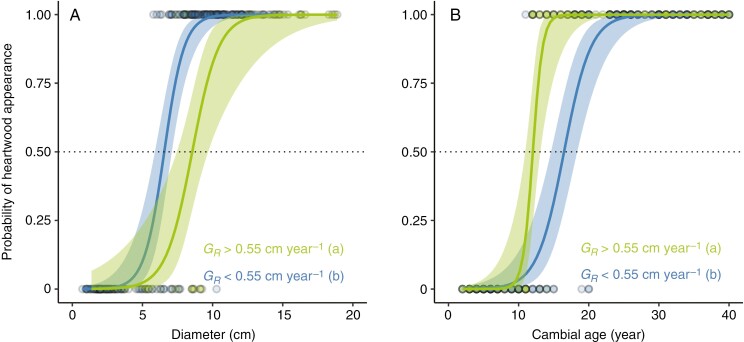
The probability of heartwood appearance in the stems of *Pericopsis elata* reveals a larger size (A) and earlier age (B) of heartwood onset in faster-growing stems (green) compared with slower-growing stems (blue). The green and blue lines show the fits of the logistic models for faster- and slower-growing stems, respectively, and the dotted line shows the 50% probability, defining the threshold size and age of heartwood onset. Two classes of growth rate (GR; in centimetres per year) were used. The significance of the difference between faster- and slower-growing stems was tested, and different letters highlight significant differences. All the discs from the trunk and from branches were included in the analysis, but the *x*-axis was shortened to highlight the curves better.

The smallest stem diameter exhibiting heartwood was 5.8 cm for shaded trees and 8.1 cm for sun-exposed trees. These stems were 11 and 12 years old, respectively, and contained one ring included in their heartwood. However, at these sizes and ages there were some stems without heartwood. In order to estimate the size and age of heartwood onset, we therefore used logistic models predicting the probability of heartwood occurrence. The models revealed significant relationships between the probability of heartwood occurrence and the stem diameter and cambial age (Fig. 3). A significant effect of growth rate was found for both the relationship between the probability of heartwood occurrence and stem diameter (Fig. 3A) and the relationship between the probability of heartwood occurrence and cambial age (Fig. 3B). Faster-growing stems showed a larger diameter (χ^2^ = 17.64; *P* < 0.001) but earlier age (χ^2^ = 16.55; *P* < 0.001) of heartwood onset compared with slower-growing stems. For a growth rate of <0.55 cm year^−1^, heartwood formation started at confidence interval (CI) 6.5 [5.7–7.3] cm and CI 15.8 [13.9–18.0] years, whereas for a growth rate of >0.55 cm year^−1^, heartwood formation started at CI 8.5 [7.8–9.9] cm and CI 12.0 [11.3–13.4] years.

### Variation and dynamics of heartwood development

**Fig. 4.**
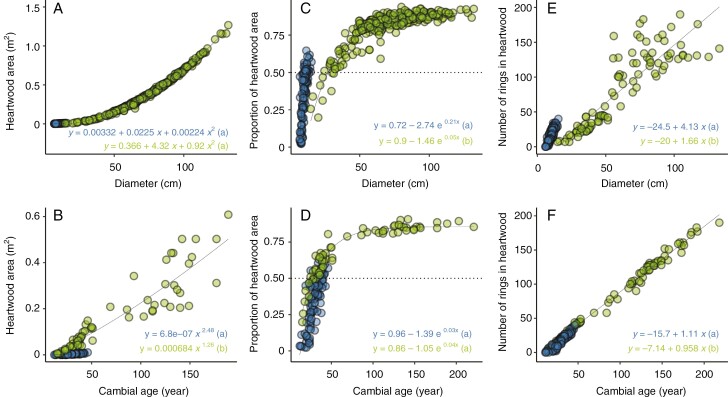
Heartwood area, proportion of heartwood area and number of growth rings included in the heartwood of *Pericopsis elata* as a function of diameter and cambial age for stems from slow-growing shaded trees (blue) and fast-growing sun-exposed trees (green). The curve and parameters of the best model used are provided for each relationship. The models used include a polynomial model ($y=a_{0}+a_{1}x+a_{2}x^{2}$; A), a power model ($y=ax^{b}$; B), a three-parameter exponential model [$y=a_{0}+\left(a_{1}-a_{0}\right)exp\left[-exp\left(\log\left(a_{2}\right)x\right)\right]$; C and D] and a linear model ($y=a_{0}+a_{1}x$; E and F). Different letters next to the model equation indicate significant differences between shaded and sun-exposed trees. In the three-parameter exponential model, the parameter $a_{0}$ is the asymptote, $a_{1}$ the intercept and $a_{2}$ the relative rate of change. All 236 discs from the trunk are included in the analysis.

Overall, heartwood area, proportion of stem area and the number of rings included in heartwood increased with stem size and age (Fig. 4). The polynomial regression best predicted the relationship between heartwood area and stem diameter (Fig. 4A), and no significant difference was found between shaded and sun-exposed trees (*P* = 0.991). However, a significant difference was found between shaded and sun-exposed trees regarding the proportion of heartwood area fitted by the three-parameter exponential regression (Fig. 4C). As the stem diameter increased, the proportion of heartwood area increased at a higher relative rate in shaded trees. For the relationship between the number of growth rings included in the heartwood and stem diameter (Fig. 4E), the linear regression showed better performance, and a significant difference was found between shaded and sun-exposed trees, with a higher number of heartwood rings per unit stem increment in shaded trees compared with sun-exposed trees.

The relationship between heartwood area and cambial age was estimated best by the power regression. A significant difference was found between stems from shaded (lower intercept with higher slope) and sun-exposed trees (higher intercept with lower slope) (Fig. 4B). For the same age, heartwood area was larger for stems from sun-exposed trees, but the relative annual rate of increase in the heartwood area was higher on stems from shaded trees. The relationship between the proportion of heartwood area and cambial age was asymptotic, given that the three-parameter exponential model performed best (Fig. 4D). Two parameters (expressing the maximum attainable percentage of heartwood area and the rate of increase in the proportion of heartwood area) showed no significant differences for stems from shaded and sun-exposed trees. However, the intercept parameter showed significant differences, with a higher intercept for stems from sun-exposed trees. Likewise, we found a significant relationship between the number of rings included in heartwood and the cambial age (Fig. 4F), and a significant difference was found between shaded and sun-exposed trees. In shaded trees, a little more than one growth ring was converted into heartwood annually, whereas in sun-exposed trees only 0.96 of a ring was converted into heartwood annually. From this relationship, the year when the first growth ring was converted into heartwood was 15 [14.6–17.3] years for shaded trees and 9 [6.63–11.66] years for sun-exposed trees.

Quantities of heartwood and sapwood also varied within tree as a function of the distance from the tree top (Table 2). From the top to the base of the tree, the radius and the cross-sectional area of stem and heartwood increased; the width of sapwood decreased, whereas its area increased. Stem, sapwood and heartwood tapering (i.e. the slope of the standardized major axis regression) differed between shaded and sun-exposed trees. The stem, in terms of both radius and area, tapered in a similar manner in shaded and sun-exposed trees (*P* = 0.659), whereas the tapering of sapwood width and heartwood radius was significantly different between shaded and sun-exposed trees (*P* < 0.001). We found that sapwood width and heartwood radius tended to taper more in shaded trees. The sapwood width of sun-exposed trees did not taper (*P *= 0.229). When observing the areas, we found that only the tapering of heartwood area differed between the two groups, with higher tapering in shaded than sun-exposed trees. In terms of proportion, a significant size effect was observed (Supplementary Data Fig. S1). The proportion of heartwood area decreased from the bottom to the top of the tree trunk, and its variation along the trunk was less pronounced as the tree became taller. In the tallest tree, the proportion of heartwood area was constant throughout the trunk.

**Table 2.** Regression coefficients, 95 % confidence intervals and significance of the standardized major axis regression used to estimate the relationship between the distance from the tree top (*L*; in metres) and the stem radius (*S*_R_; in centimetres), sapwood radius (SW_R_; in centimetres), heartwood radius (HW_R_; in centimetres), stem cross-sectional area (CSa; in centimetres squared), sapwood area (SWa; in centimetres squared), heartwood area (HWa; in centimetres squared), hydraulically weighted vessel diameter (*D*_H_; in micrometres) and total conductive vessel area (TCVA; in centimetres squared) of *Pericopsis elata* in shaded and unshaded environments

Predictor	Shaded trees			Sun-exposed trees		
	Intercept	Slope	*R*²	Intercept	Slope	*R*²
Stem, sapwood and heartwood						
*S*_R_ vs. *L*	−0.04 [−0.16 to 0.09]	0.67 [0.62–0.72]^a^	0.83	0.97 [0.54–1.39]	0.63 [0.50–0.80]^a^	0.57
SW_R_ vs. *L*	2.75 [2.45–3.05]	−0.87 [−1.00 to −0.75]^a^	0.40	2.42 [2.03–2.81]	−0.38 [−0.54 to −0.27]^b^	0.04
HW_R_ vs. *L*	−3.83 [−4.36 to −3.31]	1.96 [1.76–2.18]^a^	0.69	−0.16 [−0.70 to 0.37]	0.91 [0.75–1.11]^b^	0.67
CSa vs. *L*	1.08 [0.83–1.32]	1.33 [1.24–1.44]^a^	0.83	3.07 [2.22–3.93]	1.26 [1.00–1.59]^a^	0.57
SWa vs. *L*	1.84 [1.56–2.13]	1.72 [1.44–2.01]^a^	0.41	3.56 [2.91–4.20]	0.75 [0.57–1.01]^a^	0.32
HWa vs. *L*	−6.27 [−7.30 to −5.24]	3.82 [3.42–4.26]^a^	0.68	0.84 [−0.25 to 1.93]	1.81 [1.48–2.23]^b^	0.66
Xylem conduit						
*D*_H_ vs. *L*	131.8 [126.4–137.3]	−2.71 [−3.20 to −2.29]^a^	0.28	100.2 [91.5–108.9]	1.23 [0.88–1.72]^b^	0.17
TCVA vs. *L*	−0.37 [−1.64 to 0.89]	0.53 [0.44–0.65]^a^	0.02	−4.55 [−27.7 to 18.6]	3.58 [2.63–4.88]^b^	0.29

All variables were ln-transformed. Different letters next to the slope indicate a significant difference between shaded and sun-exposed trees.

In addition to this divergent tapering of heartwood between shaded and sun-exposed trees, a divergent tapering of xylem conduits was also identified (Table 2). As the distance from the tree top increased, *D*_H_ decreased in shaded trees (*R*^2^ = 0.37; *P* < 0.001) but increased and levelled off in sun-exposed trees (*R*^2^ = 0.29; *P* = 0.008). However, TCVA of stems increases significantly in sun-exposed trees (*R*^2^ = 0.29; *P* = 0.001) and did not vary in shaded trees (*R*^2^ = 0.02; *P* = 0.114). At the tree level, we found a mean conduit tapering of 0.15 [0.11–0.21] (*R*^2^ = 0.24; *P* = 0.005) for sun-exposed trees and −0.29 [−0.34 to −0.24] (*R*^2^ = 0.22; *P* < 0.001) for shaded trees.

### Drivers of heartwood development

**Fig. 5.**
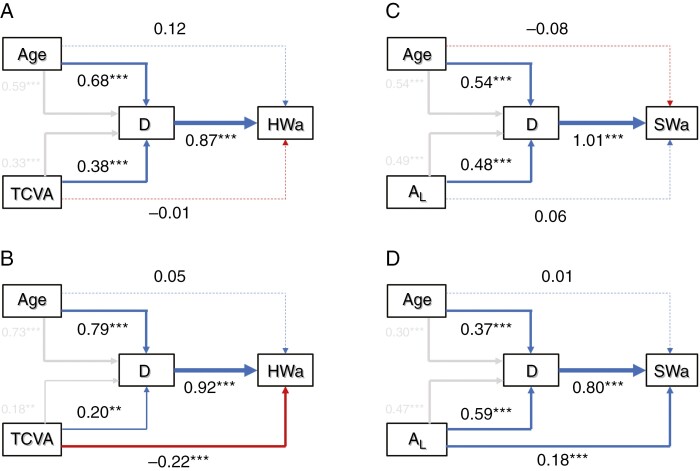
Structural equation models highlighting the direct (blue for positive and red for negative) and indirect (grey) effects of stem age (*Age*; in years) and the total conductive vessel area (TCVA; in centimetres squared) on the heartwood area (HWa; in centimetres squared) of *Pericopsis elata* for stems along the trunk of sun-exposed trees (A) and shaded trees (B). The direct and indirect effects of age and total leaf area (*A*_L_; in centimetres squared) on the sapwood area (SWa; in centimetres squared) of branches are also provided for sun-exposed trees (C) and shaded trees (D). The standardized path coefficients are provided for the direct effects of both variables and their indirect effect through stem diameter (*D*). The significance of effects is indicated as follows: **P < 0.01; and ***P < 0.001.

Structural equation models confirmed the significant effect of stem diameter on heartwood area for stems from both shaded and sun-exposed trees (Fig. 5). However, the effect of age and TCVA on heartwood area was different in shaded and sun-exposed trees. For stems from sun-exposed trees, no direct effect was found between age and heartwood area (standardized coefficient [Std. coef.] = 0.12; *P* = 0.285) or between TCVA and heartwood area (Std. coef. = −0.01; *P* = 0.893). Both variables had a significant indirect effect on heartwood area through stem diameter. Despite the high indirect effect of age (Std. coef. = 0.59; *P* < 0.001) compared with TCVA (Std. coef. = 0.33; *P* < 0.001) on heartwood area, the difference between both indirect effects was not significant (Wald χ^2 ^= 1.28; *P* = 0.258).

For stems from shaded trees, a significant direct effect of TCVA on heartwood area was observed (Std. coef. = −0.22; *P* < 0.001). However, the direct effect of age on heartwood area was insignificant (Std. coef. = 0.05; *P* = 0.223). In addition to the direct effects, significant indirect effects were observed for age on heartwood area (Std. coef. = 0.73; *P* < 0.001) and for TCVA on heartwood area (Std. coef. = 0.18; *P* = 0.003) through stem diameter. The direct effect of TCVA on heartwood area was significantly different from the indirect effect of TCVA and heartwood area through stem diameter (Wald χ^2^ = 35.44; *P* < 0.001). The direct effect was higher than the indirect effect. The combination of direct and indirect effects of TCVA on heartwood area showed no significant difference from the indirect effect of age through stem diameter on heartwood area (Wald χ^2^ = 2.08; *P* = 0.149). A very similar pattern was observed at the branch level.

For branches from sun-exposed trees, we found no direct effect of age (Std. coef. = −0.084; *P* = 0.106) and total leaf area (Std. coef. = 0.061; *P* = 0.217) on the sapwood area. However, both variables had a significant indirect effect on sapwood area. The indirect effect of age (Std. coef. = 0.54; *P* < 0.001) on sapwood area was significantly higher than the indirect effect of total leaf area (Std. coef. = 0.49; *P* < 0.001) on sapwood area (Wald χ^2^ = 38.69; *P* < 0.001). For branches from shaded trees, a significant direct effect of total leaf area on sapwood area was observed (Std. coef. = 0.18; *P* = 0.001). However, we found a non-significant direct effect of age on sapwood area (Std. coef. = 0.013; *P* = 0.781). Additionally, we found significant indirect effects for both age (Std. coef. = 0.30; *P* < 0.001) and total leaf area on sapwood area (Std. coef. = 0.47; *P* < 0.001) through stem diameter. The combination of direct and indirect effects of total leaf area on heartwood area showed no significant difference from the indirect effect of age through stem diameter on sapwood area (Wald χ^2^ = 0.90; *P* = 0.342).

## DISCUSSION

The aim of this study was to investigate the formation of heartwood in *P. elata*, an endangered and valuable timber species of the Congo Basin. We determined the age of heartwood onset and the rate of heartwood formation, and we disentangled the drivers of this process through structural equation modelling, examining the ageing and hydraulic effects on the quantity of heartwood. Growth rate was decisive in the timing of heartwood formation. Faster-growing trees produced heartwood earlier but more slowly than slower-growing trees. Tree age and hydraulic architecture mutually affected the amount of heartwood in *P. elata*, with a prominent role of tree hydraulics in the dynamics of heartwood formation, especially in limited growing conditions.

### *Early heartwood onset in faster-growing stems of* P. elata

**Fig. 6.**
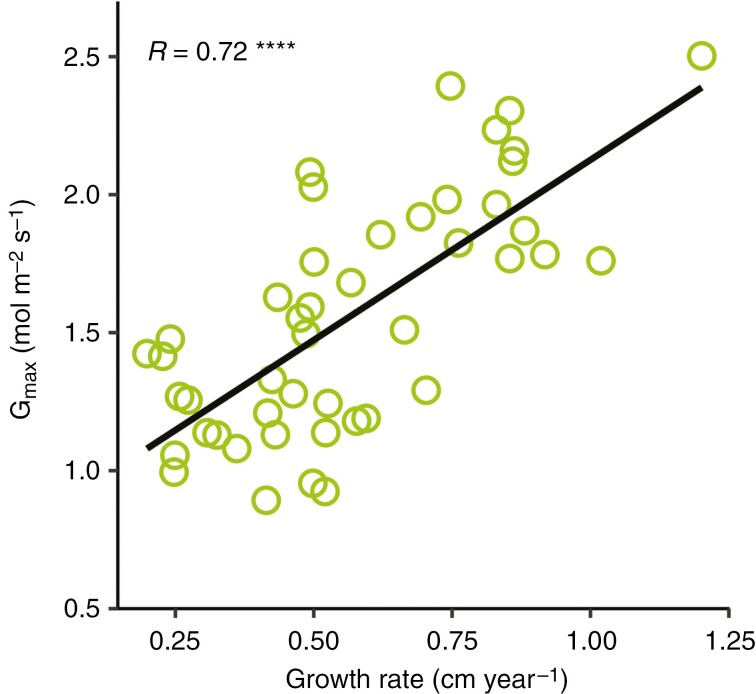
Relationship between the maximum stomatal conductance and growth rate of branches of *Pericopsis elata*. The correlation coefficient and the significance of the relationship are provided. Significance is indicated with *** for *P* < 0.0001.

We tested the effect of growth rate on the onset age of heartwood formation. We found that faster-growing stems of *P. elata* were early producers of heartwood. Hillis (1987) reported that the formation of heartwood can be influenced by the environment and forest practices, suggesting that initiation of heartwood can be delayed at poor sites. Although the effect of growth rate on the timing of heartwood onset has not been tested in previous studies, other results in literature support this finding. In *Pinus sylvestris*, it was found that trees with large crowns and growing in warmer and drier regions delayed their heartwood formation. The higher water demand of the larger crowns associated with the lower water availability in the drier region might have induced hydric stress, leading to a reduced growth rate. Consequently, the delayed heartwood onset of *Pinus sylvestris* in drier regions is associated with the reduced growth of this species in dry regions. This assumption is consistent with the results of Taeger *et al.* (2013), who found a reduced growth rate of *Pinus sylvestris* in drier regions. The effect of tree growth on the timing of heartwood onset is more likely to be triggered by the hydraulic architecture than by age. In this study, we found a significant positive effect of the maximum stomatal conductance on growth rate (Fig. 6), whereas the effect of age was non-significant. The increasingly higher annual investment in leaf area, associated with an optimal tapering of xylem conduits, resulted in faster growth of sun-exposed trees. This result suggests that the earlier heartwood formation onset of fast-growing trees is promoted by their hydraulic architecture through the positive effect of hydraulic conductivity on tree growth, as reported by Fan *et al.* (2012). However, the hydraulic variables that trigger heartwood initiation are yet to be quantified.

### *Faster heartwood investment in slower-growing stems of* P. elata

When heartwood formation is initiated, its dynamics are positively related to size and age. We found significant positive relationships between heartwood quantities (heartwood area, proportion of heartwood area and number of growth rings included in heartwood) and stem diameter and cambial age, as reported in previous research (Climent *et al.*, 2003; Pinto *et al.*, 2004; Lehnebach *et al.*, 2017). The relationship with cambial age expresses the heartwood dynamics over time. We showed that the quantity of heartwood increases as the stem ages. For the same age, faster-growing sun-exposed trees showed larger heartwood areas than slower-growing shaded trees. However, the slope of the power relationship between heartwood area and cambial age was 2.6 times higher in shaded trees than sun-exposed trees, suggesting a higher heartwood investment in shaded trees compared with sun-exposed trees. This slow heartwood investment by sun-exposed trees can be associated with the need for a larger sapwood area to store more water and photosynthates, necessary to sustain their higher growth and buffer the hydric stress imposed by their greater height and larger crown (Knapic and Pereira, 2005; van der Sande *et al.*, 2015; Lehnebach *et al.*, 2017). The larger sapwood area for sun-exposed trees might obey an allometric relationship between total leaf area and sapwood area. We found evidence to support this. The significantly negative relationship between the rate of heartwood production and the maximum stomatal conductance suggests that trees with a higher water demand at the crown slow down their heartwood production. However, the power relationship between heartwood area and cambial age also reveals a significantly lower intercept for shaded trees compared with sun-exposed trees. The lower intercept suggests that for the same age, the heartwood area of shaded trees is significantly smaller than for sun-exposed trees. This size effect, which is attributable to differences in growth rate, results in a higher absolute heartwood area in sun-exposed compared with shaded trees.

Within trees, we found that despite a similar tapering of stem and sapwood area between shaded and sun-exposed trees, the heartwood area was more tapered in shaded trees (Table 2). This is attributable to the size effect, especially tree height. The negative relationship between tree height and heartwood tapering suggests that the heartwood of *P. elata* becomes less tapered when trees grow taller. The lower total height potentially limits the heartwood in shaded trees to reach a lower maximum height (Gominho and Pereira, 2005; Knapic *et al.*, 2006; Morais and Pereira, 2007; Flæte and Høibø, 2009). Given that the height of *P. elata* at the studied site approached an asymptotic value when the stem diameter was ~49.21 [46.98–51.44] cm (Kafuti *et al.*, 2022), we expect that the heartwood tapering of the species would reach an asymptote when this stem diameter is reached. Consistent results have been found in this study. From this diameter onwards, heartwood tapering was negligible (Supplementary Data Fig. S1) and the rate of heartwood rings production approaches a value of one ring per year. This means that from this diameter onwards, heartwood forms at a similar rate to stem increment, and almost the total area of stem increment is converted into heartwood. The saturation of heartwood production occurring concomitantly with the saturation of tree height supports the hydraulic assumption of heartwood production (Tyree and Zimmermann, 2002). As tree grows taller, the hydraulic resistance increases, and a certain amount of sapwood is needed to compensate for the hydric stress resulting from increasing tree height. However, when the growth in tree height levels off, the path-length hydraulic resistance does not increase any further, and as a result, the buffering role of additional sapwood area is no longer needed. Consequently, for a given area of stem increment, a similar area of sapwood is converted into heartwood.

### *Heartwood dynamics in* P. elata *are related to both ageing and hydraulics*

We have shown that heartwood quantities are related tightly to stem diameter. The effect of size on the quantity of heartwood includes not only a hydraulic and metabolic component, owing to the increasing hydraulic requirements and maintenance cost associated with the size increase (Lachenbruch and McCulloh, 2014; McCulloh *et al.*, 2015), but also an age effect, as a result of an increase in size as the tree ages (O’Brien *et al.*, 1995). Disentangling the hydraulic effect and the age effect was challenging. Despite an increasing number of studies reporting a positive relationship between heartwood quantities and stem age (Yang and Hazenberg, 1991*b*; Sellin, 1994; Climent *et al.*, 2002), the effect of age on the quantity of heartwood is obscured by stem diameter when modelled together (Moya and Perez, 2008; Moya *et al.*, 2014).

We therefore developed a theoretical model whereby age and hydraulic-related variables can be related directly to heartwood area or indirectly, through stem diameter. We tested this using structural equation models and found that despite the large effect of hydraulic variables on heartwood and sapwood quantities, the effect of age was not negligible. The hydraulic-related variables showed significant direct and indirect effects on heartwood and sapwood quantities, whereas the effect of age was essentially indirect (i.e. through stem diameter) but significant (Fig. 5). At the trunk level, the hydraulic-related variable TCVA (expressing the ability to supply water) was negatively related to the heartwood area, while at the branch level the hydraulic-related variable total leaf area (expressing the water demand) was positively related to the sapwood area. This suggests that stems with higher water demand or higher ability to supply water need more sapwood and less heartwood.

This is consistent with the hydraulic hypothesis suggesting that heartwood formation is a mechanism controlling the amount and the longevity of sapwood to an optimum level to an optimum level for the transport of sap and the storage of carbohydrates or nutrients (Bamber, 1976; Moreno Chan *et al.*, 2013; Castorena *et al.*, 2022). Besides this effect of hydraulic-related variables, we also found that stem ageing results in an increase in the heartwood and sapwood area. This result aligns with the assumption of an internal ‘timer’ triggering physiological transformations in trees (Gjerdrum, 2003). In sun-exposed trees, no direct effect of either ageing or hydraulic-related variables was found, whereas in shaded trees we found a significant direct effect of hydraulic-related variables on heartwood and sapwood area. This suggests a shared effect of age and tree hydraulics on the dynamics of heartwood formation in *P. elata*. In limiting conditions, where trees exhibit a non-optimal hydraulic architecture, the dynamics of heartwood formation are driven much more by hydraulic variables.

## SUPPLEMENTARY DATA

Supplementary data are available at *Annals of Botany* online and consist of the following.

Figure S1: Proportion of heartwood area along the trunk of small, medium and large trees.

mcad079_suppl_Supplementary_Material
