# Supplementary material for: Earlier onset and slower heartwood investment in faster-growing trees of African tropical species
Source: Ann Bot. 2023 Jul 6;133(5-6):905–16. doi: 10.1093/aob/mcad079 (PMC11082515; doi:10.1093/aob/mcad079)
Supplement: mcad079_suppl_Supplementary_Material [file mcad079_suppl_supplementary_material.zip › aob-23138-s07.docx]

*Supplementary material*

**Earlier onset and slower heartwood investment in faster-growing trees of African tropical species**

**Chadrack Kafuti ^1,2,3,^*, Romain Lehnebach ^1,4^, Nils Bourland ^2,5^, Hans Beeckman ^2^, Joris Van Acker ^1^, Nestor Luambua ^2,6^ and Jan Van den Bulcke ^1^**

*^1^ UGent-Woodlab, Laboratory of Wood Technology, Department of Environment, Ghent University, Coupure Links 653, 9000 Gent, Belgium, ^2^ Service of Wood Biology, Royal Museum for Central Africa, Leuvensesteenweg 13, 3080 Tervuren, Belgium, ^3^ Faculty of Agricultural Sciences, Department of Natural Resources Management, University of Kinshasa, 117 Kinshasa XI, Democratic Republic of the Congo, ^4^ CIRAD, UMR Ecologie des Forêts de Guyane (EcoFoG), AgroParisTech, CNRS, INRA, Université Des Antilles, Université de Guyane, 97310 Kourou, France, ^5^ Center for International Forestry Research, Situ Gede, Sindang Barang, Bogor (Barat) 16115, Indonesia, ^6^ Faculté des sciences Agronomiques, Université Officielle de Mbujimayi, Mbujimayi, Democratic Republic of Congo*

Running title: Dynamics and drivers of heartwood formation in African tropical trees

** For correspondence. E-mail:* [*chadrack.kafuti@ugent.be*](mailto:chadrack.kafuti@ugent.be)

**APPENDIX**

**Fig.A1** Proportion of heartwood area along the trunk of small (blue, this corresponds to the shaded trees), medium (white, this corresponds to the sun-exposed trees) and large trees (green, this corresponds to all the other trees from the natural unmanaged forest). The significant decrease in the heartwood tapering is clear, with small, medium and large trees having a diameter from 9 to 15 cm, from 25 to 40 cm and from 50 to 150 cm respectively

**
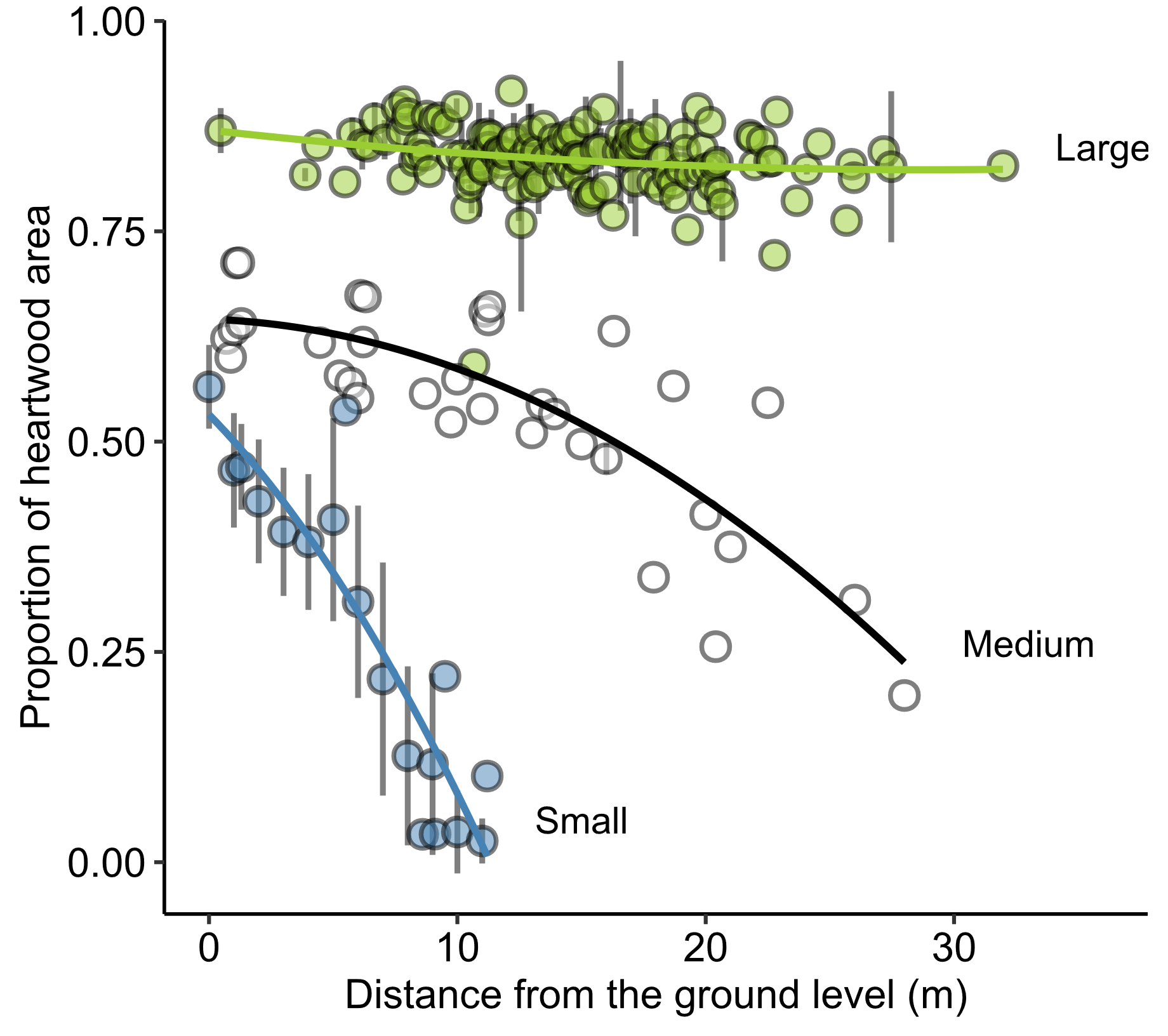
**
