# Supplementary figures and images for: Earlier onset and slower heartwood investment in faster-growing trees of African tropical species
Source: Ann Bot. 2023 Jul 6;133(5-6):905–16. doi: 10.1093/aob/mcad079 (PMC11082515; doi:10.1093/aob/mcad079)

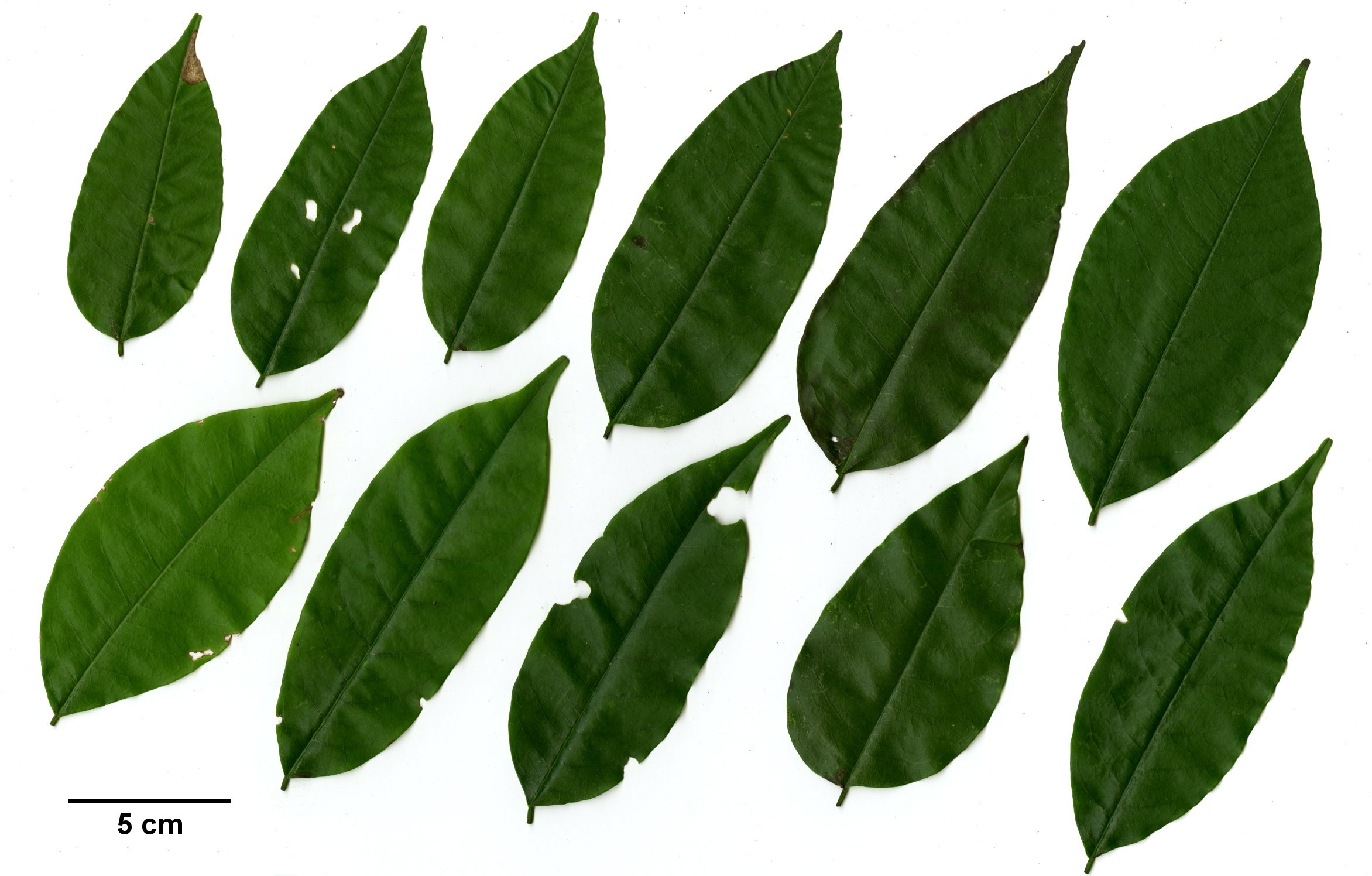

Supplement: mcad079_suppl_Supplementary_Material [file mcad079_suppl_supplementary_material.zip › aob-23138-s01.jpg]

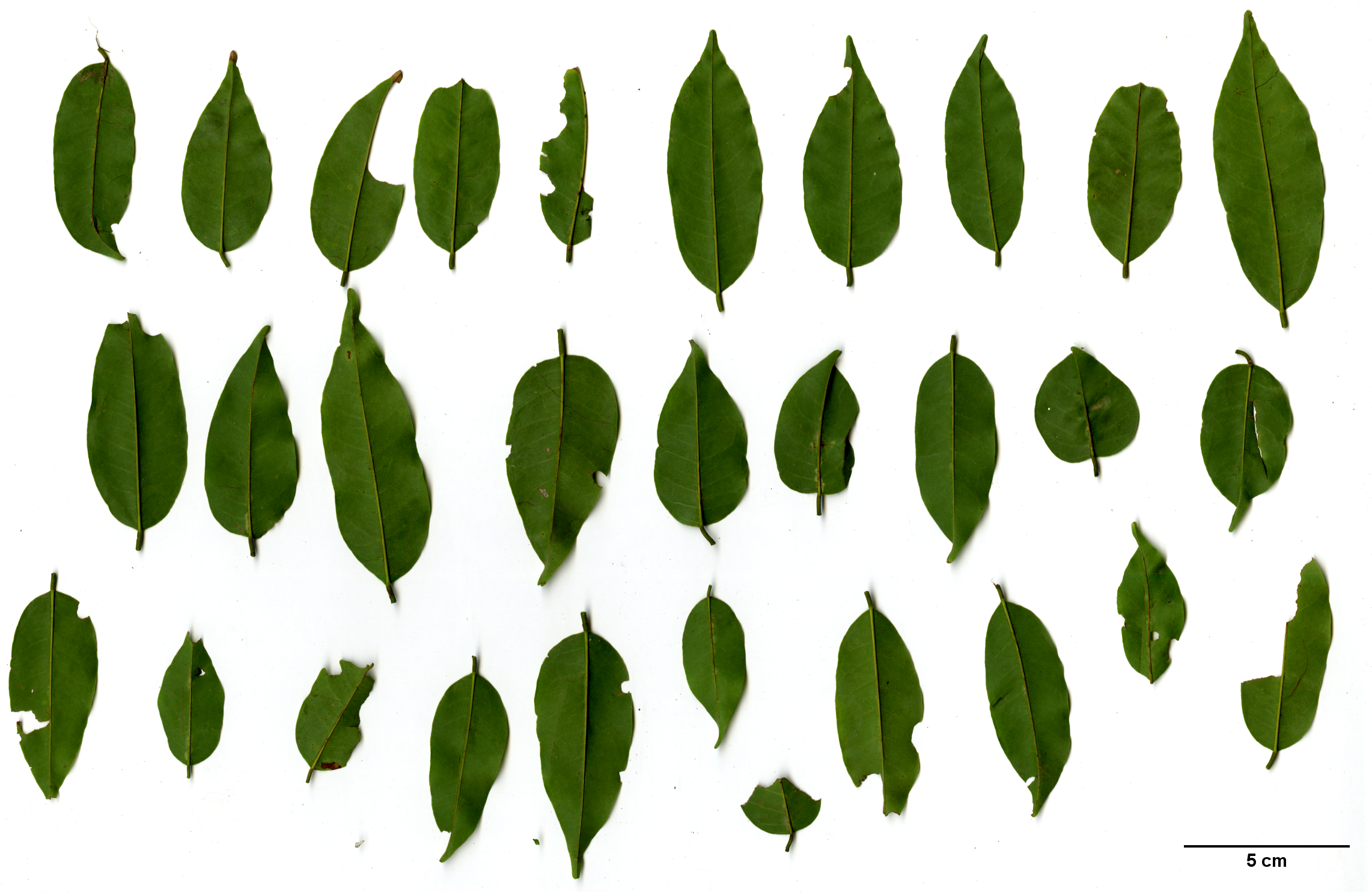

Supplement: mcad079_suppl_Supplementary_Material [file mcad079_suppl_supplementary_material.zip › aob-23138-s02.png]

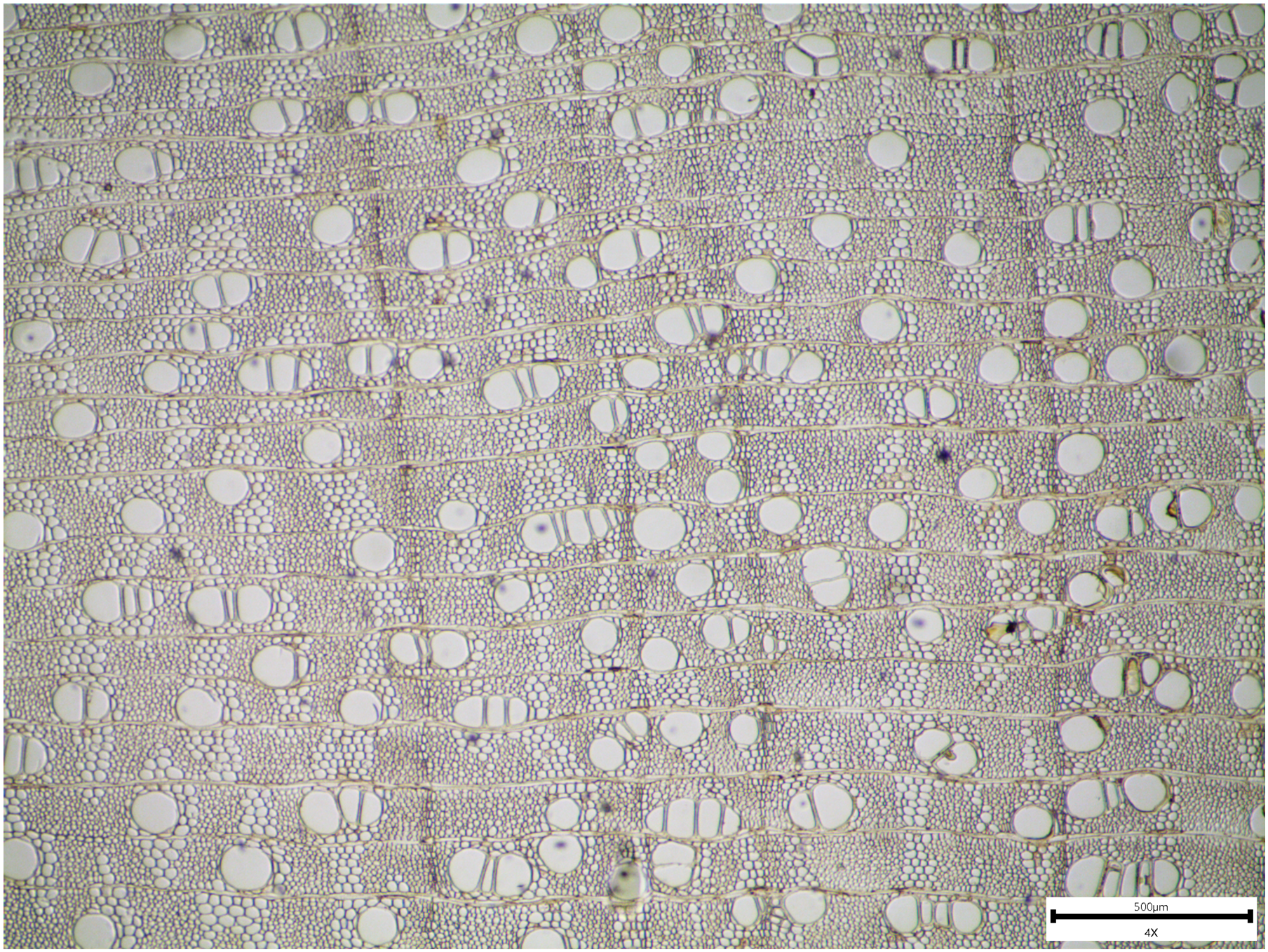

Supplement: mcad079_suppl_Supplementary_Material [file mcad079_suppl_supplementary_material.zip › aob-23138-s03.png]

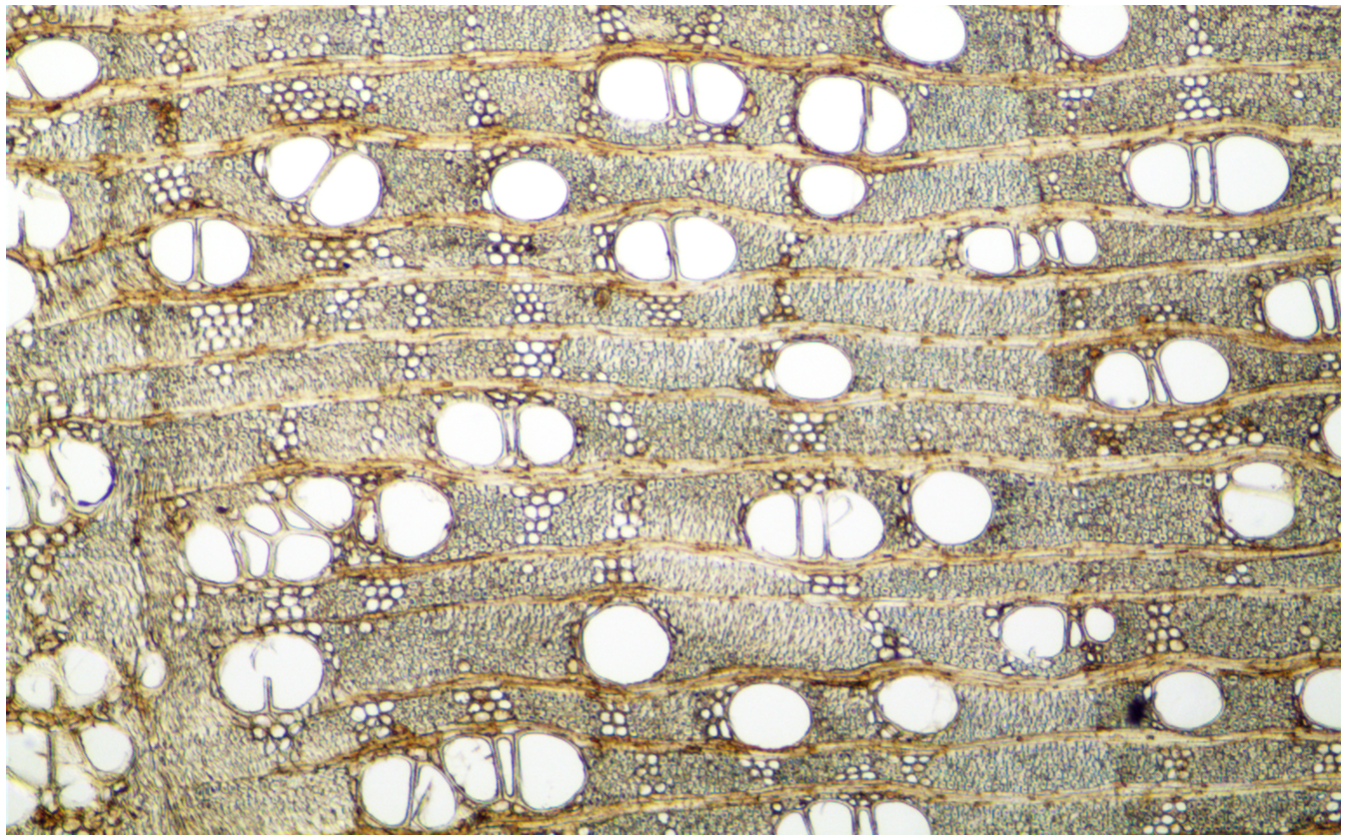

Supplement: mcad079_suppl_Supplementary_Material [file mcad079_suppl_supplementary_material.zip › aob-23138-s04.png]

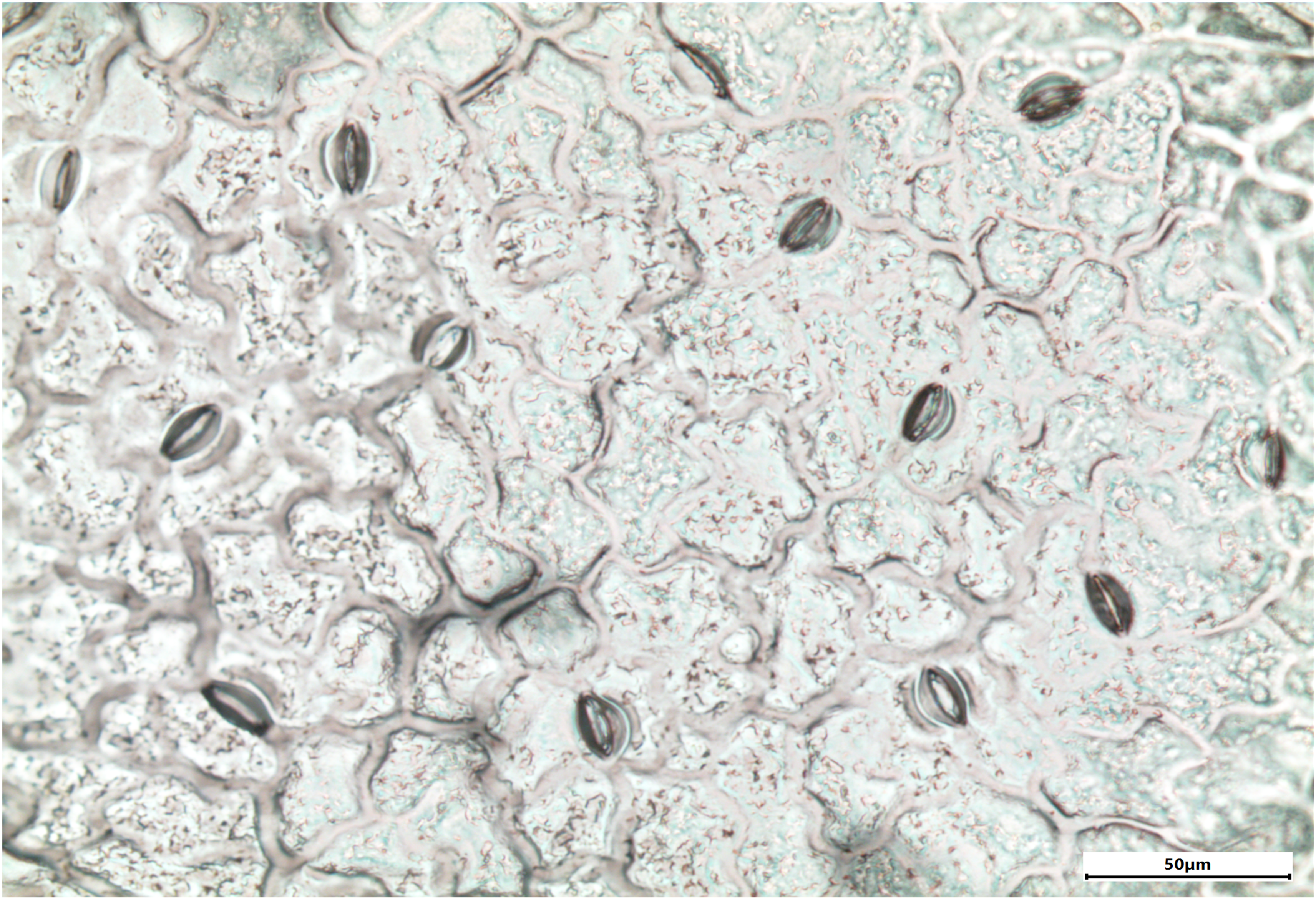

Supplement: mcad079_suppl_Supplementary_Material [file mcad079_suppl_supplementary_material.zip › aob-23138-s05.png]

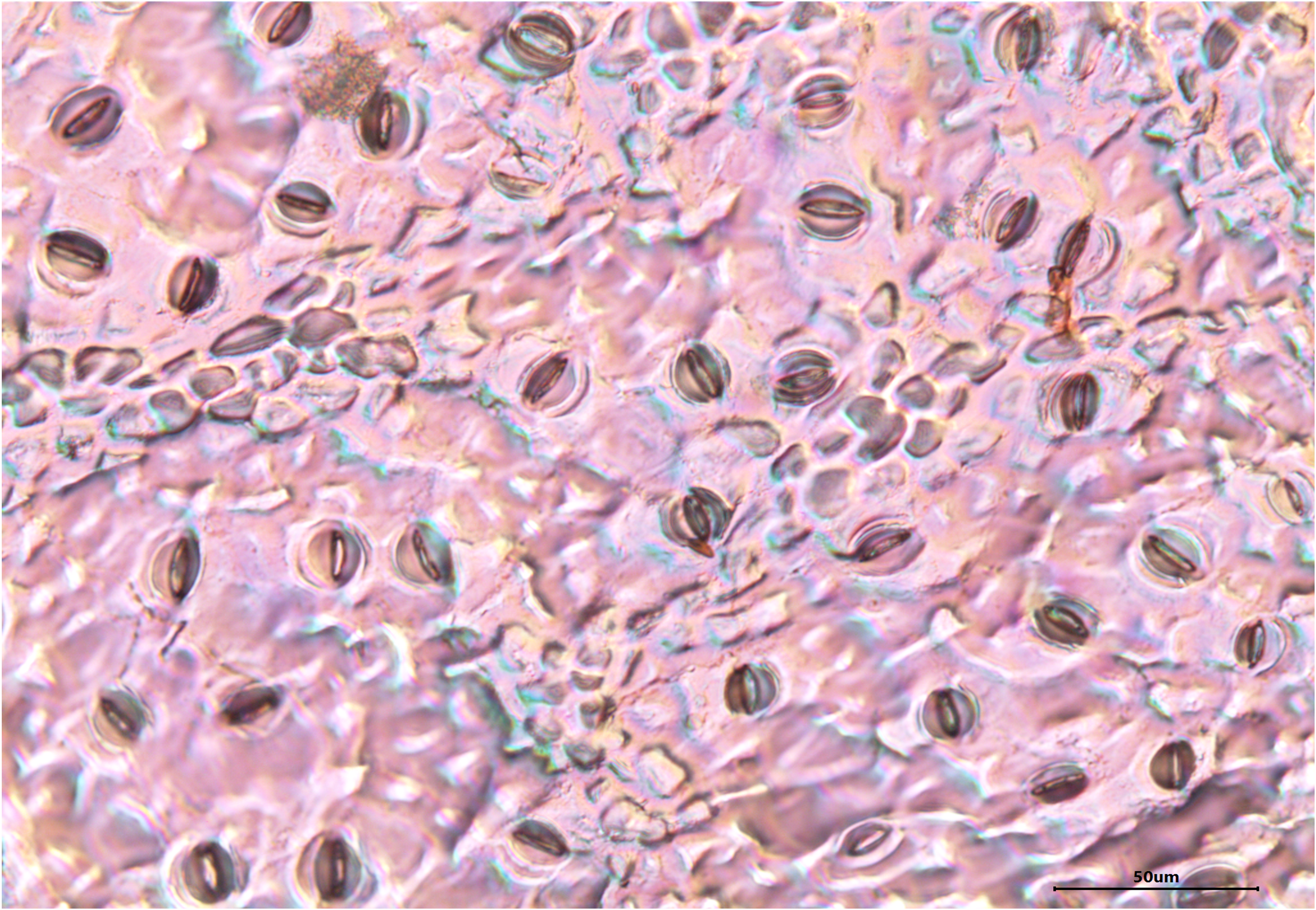

Supplement: mcad079_suppl_Supplementary_Material [file mcad079_suppl_supplementary_material.zip › aob-23138-s06.png]
